# *Para-*phenylenediamines and their oxidative derivatives in infant formulas in China: Occurrence and health implications

**DOI:** 10.1016/j.fochx.2026.104100

**Published:** 2026-06-13

**Authors:** Zifu Shao, Chunyan Gao, Zhixiong Shi

**Affiliations:** a Yanjing Medical College, Capital Medical University, Beijing 101300, China; b School of Public Health, Capital Medical University, Beijing 100069, China

**Keywords:** *Para*-phenylenediamines, Infant formula, Exposure assessment, Health risks, PPD-quinone

## Abstract

*Para-*phenylenediamines (PPDs) and their quinone derivatives (PPD-Qs) are ubiquitous in the environment, while data on their occurrence in foods are scarce. In this study, twelve PPDs and PPD-Qs were measured in infant formula, and their daily intakes and health risks for infants were assessed. In the tested 141 samples, the median levels of ∑PPDs and ∑PPD-Qs were 2538 and 80.34 pg/g (powder), respectively. N-(1,3-Dimethylbutyl)-*N*′-phenyl-*p*-phenylenediamine (6PPD) was detected in most samples, with a median level of 2125 pg/g. However, most other PPDs and all PPD-Qs presented low detection frequencies of <40%, with mean levels of <80 pg/g. Formulas for infants of 0–6 months had significantly higher levels of 6PPD than those for older infants. Estimated daily intakes (EDIs) of ∑PPDs and ∑PPD-Qs via formula feeding were < 200 and < 10 ng/kg bw/day, respectively, for infants, and formula feeding may represent an important dietary exposure pathway for infants.

## Highlights

• Twelve PPDs and PPD-Qs were measured in infant formulas from China
• 6PPD was widely detected and was the dominant PPD in infant formulas
• Most other PPDs and all PPD-Qs showed low detection frequencies
• First-stage formulas contained higher 6PPD levels than formulas for older infants
• Formula feeding may represent an important dietary exposure pathway for infants

## 1 Introduction

The discovery of urban runoff mortality syndrome affecting Pacific Northwest coho salmon has drawn global attention to N-(1, 3-dimethylbutyl)-*N*′-phenyl-*p*-phenylenediamine (6PPD) and its analogues within the N, N′-substituted *p*-phenylenediamine (PPD) antioxidant class (Tian et al., 2021; Wang et al., 2026). *Para-*phenylenediamines (PPDs), a group of organic compounds primarily used as rubber antioxidants, are now under increasing scrutiny. China has emerged as the world's leading producer and supplier of PPDs, and widespread use of these chemicals has led to their release into the environment. For example, the global release of tire wear particles (TWPs) containing residual 6PPD has exceeded 330 tons per year, at least 300 tons of which are deposited into soil (Yi et al., 2025). TWPs, generated as tires rub against road surfaces, can release PPDs to the environment through rain runoff or evaporation, making them the major source of PPDs in the environment. Moreover, large amounts of discarded tires can also leach PPDs into surrounding ecosystems. Upon exposure to ozone, PPDs can readily transform into their quinone derivatives (PPD-Qs) (Zhao et al., 2023). As a result, PPDs and PPD-Qs are widespread in various environmental matrices, including soil (Cao et al., 2022), atmospheric particles (Wang et al., 2022; Zhang et al., 2022), water (Johannessen & Metcalfe, 2022; Zhu et al., 2024), sediment (Zeng et al., 2023), and dust (Zhang, Cheng, et al., 2024).

Currently, data on both toxicity and environmental pollution of PPDs and PPD-Qs remain limited, with most studies focusing only on 6PPD and 6PPD-Q. 6PPD-Q, a transformation product of 6PPD, is a cause of acute mortality in coho salmon (Tian et al., 2022). However, recent studies show that exposure to 6PPD-Q even at low concentrations can exert adverse effects on the nervous system, growth and development, and metabolism of aquatic organisms (Chae et al., 2026; Hua et al., 2023). Besides the threat to aquatic life, 6PPD and 6PPD-Q may also pose health risks to mammals, such as reproductive and developmental toxicity (Matsumoto et al., 2013; Yao et al., 2024), hepatotoxicity (Fang et al., 2023), and intestinal toxicity (Yang et al., 2024).

Since PPDs and PPD-Qs are frequently detected in the environment, concerns about their occurrence in biota and humans and potential adverse effects have risen sharply. It has been confirmed that PPDs/PPD-Qs can enter the food chain through soil, water, and atmospheric deposition (Wei et al., 2024), and they have been detected in fruits, vegetables, and some aquatic foods (Breider et al., 2025; Ji et al., 2022; Sartori et al., 2026; Zhang, Wang, et al., 2024). Investigations into exposure pathways suggest that neither dust ingestion nor dermal exposure is the main intake pathway of PPDs and PPD-Qs for the general population, and intake via foods (including water) might be a main source (Guo et al., 2024). However, data on their presence in foodstuffs are still scarce. As chemical pollutants, PPDs and PPD-Qs can accumulate in the human body through multiple pathways, i.e., dust ingestion, dietary intake, and dermal exposure, and may pose potential health risks (Song et al., 2024; Zhang, Cheng, et al., 2024). For example, Song et al. provided critical insights into human exposure and hepatotoxicity, demonstrating a positive correlation between 6PPD concentrations in serum and oxidative stress biomarkers in both healthy individuals and patients with nonalcoholic fatty liver disease (Song et al., 2024).

Human milk is well known as the best food for newborns, while PPDs and PPD-Qs have been detected in human milk, raising concerns about their potential health risks to newborns (Liang et al., 2024; Wu et al., 2024). However, the limited available human-milk studies have reported markedly different occurrence patterns of PPDs and PPD-Qs. Besides human milk, infant formula serves as another important food source for newborns and infants due to low breastfeeding rates in China. A survey conducted in Southern China reported that the exclusive breastfeeding rate for 6 months or more was only 42.26%, which was even lower than a previous survey conducted in 2013 (Zhang et al., 2025). That is, at least half of the infants may require formula feeding because of insufficient breast milk or other reasons. However, no study has reported the presence of PPDs and PPD-Qs in infant formula and associated risks to newborns and infants. Moreover, the potential routes by which PPDs and PPD-Qs enter infant formula remain unclear. Possible sources include environmental transfer into raw milk through dairy cattle exposure to feed, drinking water, dust, or farm environments, contamination of lipid-rich ingredients in formulas, and contact with rubber-containing materials during processing or packaging.

Newborns and infants are particularly vulnerable to chemical pollutants due to their critical developmental stages. Although human and mammalian evidence is limited, zebrafish embryos exposed to low doses of 6PPD and 6PPD-Q have exhibited developmental deformities, including changes in eye size and altered gene expression related to growth and thyroid function (Peng et al., 2022; Varshney et al., 2022; Zhang et al., 2023). Meanwhile, monitoring infant formula is of public-health importance because formula can be the sole or major food source for infants who are not exclusively breastfed. Infants also have higher food intake per unit body weight, immature metabolic and detoxification capacity, and critical developmental vulnerability. Therefore, even low-level contamination in formula may contribute meaningfully to infant exposure. Since the occurrence of PPDs and PPD-Qs in infant formula, as well as in its major ingredient, cow milk, has not been investigated, the present study aims to measure currently used PPDs and their paired PPD-Qs in commercially available infant formulas in China, and further assess the associated health risks for infants and newborns caused by the intake of PPDs and PPD-Qs via infant formula feeding.

## 2 Materials and methods

1.1. Chemicals and Reagents

Six PPDs and their paired PPD-Qs were included in the measurement. Their information, including abbreviations, full names, and CAS numbers, is listed in Table 1. Standards of the analytes were provided by Alta Scientific (Tianjin, China). Isotopically labelled d_5_–6PPD (in acetonitrile, 100 μg/mL) and d_5_–6PPD-Q (in acetonitrile, 100 μg/mL) were also from Alta Scientific Co., Ltd. (Tianjin, China). Mass spectrometry-grade methanol and acetonitrile, and HPLC-grade ammonium acetate were purchased from Fisher Scientific (Fair Lawn, NJ, USA) and J&K Chemical Ltd. (Beijing, China). *Tert*-butylhydroquinone (TBHQ), 3, 5, di-tert-butylphenol (DBP), and l-ascorbic acid (L-AA) were from Aladdin Biochemical Technology Co., Ltd. (Shanghai, China). The protective agent was prepared by dissolving 1 g each of TBHQ, DBP, and L-AA in 100 mL of acetonitrile: water (1:1, *V*/V). Ultrapure water was produced by a Milli-Q (Merck, Billerica, MA, USA). A Bond quick, easy, cheap, effective, rugged, and safe (QuEChERS) extraction salt packet (Part No. 5982–0032) containing 4 g of anhydrous MgSO_4_ and 1 g of NaCl was used to assist salting-out extraction, and the extract was further purified using a Captiva EMR-Lipid solid-phase extraction (SPE) cartridge (Part No. 5982–1003, 3 mL, 300 mg). Both salt packets and SPE cartridges were provided by Agilent Technologies (Santa Clara, CA, USA).

1.2. Sample Collection

**Table 1:** Information of PPDs and PPD-Qs tested in this study.

Compound	Full name	CAS	LOD (pg/g)	LOQ (pg/g)	Pre	Pro	FV (V)	CE (V)
IPPD	N-isopropyl-*N*′-phenyl-*p*-phenylenediamine	101–72-4	16.7	50.1	227.1	184*, 107	80	18
DPPD	*N*,*N*′-Diphenyl-*p*-phenylenediamine	74–31-7	33.3	99.9	261.1	184*, 169	120	30
6PPD	N-(1,3-Dimethylbutyl)-*N*′-phenyl-*p*-phenylenediamine	793–24-8	16.7	50.1	269.2	184*, 93	90	25
77PD	*N*,*N*′-bis(1,4-dimethylpentyl)-pphenylenediamine	3081-14-9	33.3	99.9	305.2	206, 135*	110	15
CPPD	N-Cyclohexyl-*N*′-phenyl-*p*-phenylenediamine	101–87-1	16.7	50.1	267.1	185*, 93	100	15
DTPD	*N*,*N*′-di-(o-tolyl)-*p*-phenylenediamine	15,017–02-4	16.7	50.1	289.2	198*, 106	130	25
IPPD-Q	N-Isopropyl-*N*′-phenyl-*p*-phenylenediamine quinone	68,054–73-9	16.7	50.1	257.1	215, 187*	110	12
DPPD-Q	*N*,*N*′-Diphenyl-*p*-phenylenediamine quinone	3421-08-7	16.7	50.1	291.1	263, 144*	110	35
6PPD-Q	N-(1,3-Dimethylbutyl)-*N*′-phenyl-*p*-phenylenediamine quinone	2,754,428–18-5	16.7	50.1	299.2	241, 215*	110	30
77PD-Q	*N*,*N*′-Bis(1,4-dimethylpentyl)-*p*-phenylendiamine quinone	2,894,124–00-4	16.7	50.1	335.2	237*, 139	110	20
CPPD-Q	N-Cyclohexyl-*N*′-phenyl-*p*-phenylenediamine quinone	68,054–78-4	33.3	99.9	297.1	215, 187*	110	15
DTPD-Q	N,N′-Di-(o-tolyl)-*p*-phenylendiamine quinone	252,950–56-4	16.7	50.1	319.1	212*, 184	110	20
d_5_–6PPD	N-(1,3-Dimethylbutyl)-*N*′-phenyl-*p*-phenylenediamine D5	–	–	–	274.2	190, 189*	90	10
d_5_–6PPD-Q	N-(1,3-Dimethylbutyl)-*N*′-phenyl-*p*-phenylenediamine Quinone D5	–	–	–	304.2	192*	110	30

Note: PPDs: *p*-phenylenediamines; PPD-Qs: *p*-phenylenediamine quinones; CAS: Chemical Abstracts Service registry number; LOD: limit of detection; LOQ: limit of quantification; Pre: precursor ion (*m/z*); Pro: product ion (*m/z*); FV: fragment voltage; CE: collision energy; *: quantitative ion; d5–6PPD and d5–6PPD-Q: deuterated internal standards.

A total of 141 infant formula samples covering 90 brands were purchased from supermarkets, baby product stores, and online food shops in 2024. Of these, 109 samples were from domestic manufacturers, and the remaining 32 were imported products. The samples included 42 first-stage formula samples (for infants aged 0–6 months), 40 s-stage (for 7–12 months), 55 third-stage (for 13–36 months), and 4 fourth-stage (for 37–72 months).

1.3. Sample Preparation and Instrumental Analysis

Two grams of a formula sample were accurately weighed into a 25 mL centrifuge tube, and 5 mL of water was added to dissolve. Then, 1 mL of the protective agent was added, and the mixture was vortexed for 3 min and stood for 10 min. Internal standard solution (50 μL, 100 μg/L) and 10 mL of acetonitrile were added and vortexed for 5 min, after which a Bond QuEChERS salt packet was added to induce salting-out and improve the transfer of the target analytes into the organic phase. The mixture was vortexed again for 5 min, then centrifuged at 4000 rpm for 10 min at 4 °C. Subsequently, 4 mL of the supernatant was transferred to another tube and mixed with 1 mL of water, and the solution was purified using a Captiva EMR-Lipid cartridge. Finally, the entire eluent was collected for instrumental analysis. Since the infant formula powder was analyzed as purchased, concentrations of the analytes are reported as pg/g (powder).

Notably, PPDs are susceptible to oxidation, and their quinone derivatives may also undergo further reactions during sample handling, and therefore antioxidant protection is necessary (Lucarini & Staedler, 2026). In our method, L-AA was used as a water-soluble reducing antioxidant, while TBHQ and DBP were used as phenolic antioxidants with good compatibility with organic and lipid-rich phases. Their use was designed to reduce oxidative transformation or degradation of PPDs/PPD-Qs during aqueous dispersion and acetonitrile extraction.

Instrumental analysis was performed using an Infinity 1290 ultraperformance liquid chromatography coupled to a 6495C triple quadruple mass spectrometer (UHPLC-MS/MS) (Agilent Technologies, Santa Clara, CA, USA). Chromatographic separation of the analytes was accomplished using a Pursuit 3 PFP column (100 × 2.0 mm, 3.0 μm, Agilent Technologies) with mobile phases composed of 2 mmol/L ammonium acetate (A) and methanol (B). The flow rate of the mobile phase was set at a constant of 0.4 mL/min, and the column temperature was maintained at 40 °C, with an injection volume of 2 μL. The eluent gradient began with 40% B for 1 min, then increased linearly to 50% B over 2 min, followed by a linear increase to 55% B over 4 min, then to 75% B over 1 min, then increased to 100% at 10 min and held for 2 min, and finally returned to 40% B and maintained for 3 min for equilibration.

The mass spectrometer was operated in positive mode with an electrospray ionization source (ESI+), and the capillary voltage was set at 3500 V. Nitrogen was used as drying gas (7 L/min) and sheath gas (11 L/min), with drying and sheath gas temperatures at 250 and 350 °C, respectively. The multiple reaction monitoring (MRM) mode was used for the identification and quantification, and detailed instrumental parameters for each analyte are listed in Table 1.

1.4. Quality Assurance and Quality Control (QA/QC)

To ensure the reliability and accuracy of the results, QA/QC procedures were applied throughout sample analysis. To minimize background contamination, plastic consumables were rinsed with methanol, and glassware was baked in a muffle furnace at 400 °C for 4 h. A procedural blank was analyzed every 10 samples to monitor possible background contamination, and no target analytes were detected in the procedural blanks. Calibration curves were constructed using a series of standard solutions with concentrations of 0.05–50 ng/mL (concentration of each IS was maintained at 5 ng/mL), and excellent linearity was obtained with coefficients (*R*^*2*^) of 0.9992–0.9999. Because the tested products were cow milk-based formulas and cow milk powder shares the major dairy matrix components, including milk proteins and lipids, spiked recovery tests were conducted by using cow milk powder as the spiking matrix, with spiking levels of 0.5 and 5 ng/g, respectively. Recoveries of the analytes ranged from 76.5% to 97.8%, with relative standard deviations (RSDs) lower than 15%, supporting the applicability of the method for screening-level occurrence assessment. However, cow milk powder is not identical to commercial infant formula as the formula contains additional ingredients. In addition, formula composition differs among stages. Therefore, potential matrix-dependent recovery differences among infant formula products and formula stages cannot be completely excluded. The limits of detection (LOD) and quantification (LOQ) were determined as signal-to-noise ratios of 3:1 and 10:1, respectively, yielding LODs of 16.7–33.3 pg/g and LOQs of 50.1–99.9 pg/g (Table 1).

Due to the limited commercial availability of isotopically labelled standards, only d_5_–6PPD and d_5_–6PPD-Q were used as internal standards. d_5_–6PPD was used to correct the six PPDs, and d_5_–6PPD-Q was for all PPD-Qs, based on structural similarity and chromatographic behavior. However, the analytes differ in physical and chemical properties, and therefore matrix effects, extraction recovery, or instrumental response may not be fully corrected for all analytes. Therefore, this internal-standard strategy may introduce systematic uncertainty. Nevertheless, satisfactory recovery and RSD results indicated that our method was suitable for occurrence screening and preliminary exposure assessment.

1.5. Data Analysis

Quantitative analysis was conducted using Agilent MassHunter Workstation software (Version 10.0). Statistical analysis was done by IBM SPSS 22.0 (Chicago, IL, USA), and *P* < 0.05 was considered significant. Data below LOD were reported as not detected (ND) or as half the LOD for statistical purposes, depending on the context.

1.6. Estimated Daily Intake

Estimated daily intakes (EDIs) (ng/kg bw/day) of PPDs and PPD-Qs via formula feeding were calculated as EDI=C_IF_ × ADC/BW. Where C_IF_ represents the concentration of each analyte in infant formula, and ADC is the average daily consumption of formula powder (g/day). BW (body weight) is the body weight of infants. According to the *Chinese Dietary Reference Intakes (Edition 2013)* provided by the Chinese Nutrition Society, the recommended average formula consumption and body weight of infants of different ages are as follows: (1) 0–6 months (formula powder intake of 99 g/day, BW of 6 kg); 7–12 months (formula powder intake of 113 g/day, BW of 9.2 kg); 13–36 months (formula powder intake of 91 g/day, BW of 12.6 kg).

It should be noted that the present EDI calculation only considered exposure via infant formula feeding, because only infant formula samples were analyzed in this study. For mixed-fed infants who consume both breast milk and infant formula, total dietary exposure should theoretically be estimated as the sum of formula-derived and breast-milk-derived intake. However, because paired breast-milk concentrations were unavailable, mixed-feeding and exclusive-breastfeeding exposure scenarios could not be calculated in this study.

## 3 Results and discussion

### 3.1 Concentrations and profiles of PPDs and PPD-Qs in infant formula

#### 3.1.1 The occurrence of 6PPD and 6PPD-Q in infant formula

The concentrations and detection frequencies (DFs) of PPDs and PPD-Qs in the 141 infant formula samples are listed in Table 2 (with non-detected value set to 1/2LOD). The levels of ∑PPDs (sum of the six PPDs) ranged from 81 to 30,960 pg/g (powder), with mean and median levels of 3975 and 2538 pg/g, respectively. The levels of ∑PPD-Qs (sum of the six PPD-Qs) ranged from 58.35 to 4451 pg/g, with a median of 80.34 pg/g. Notably, because most PPD-Qs had low DFs, ∑PPD-Q concentrations were sensitive to the treatment of non-detected values. For example, when non-detected values were set as 0 and LOD, the P25/P50 values of ∑PPD-Q were 15.79/31.48 and 100.2/125.48 pg/g. This large discrepancy suggested that ∑PPD-Q results partly reflect the substitution method rather than measured concentrations in all samples. Therefore, results for analytes with low DFs and summed PPD-Qs should be interpreted as screening-level estimates.

**Table 2:** Concentration of PPDs and PPD-Qs in infant formula (pg/g powder, *n* = 141).

Compounds	DF (%)	Mean	SD	P25	P50	P75	P95	Max
6PPD	97	3479	4447	264.3	2125	5108	10,560	28,560
IPPD	74	378.5	1216	<LOD	79.79	248	1333	12,830
DTPD	38	42.41	129.26	<LOD	<LOD	26.7	137.4	1311
DPPD	9	20.22	38.42	<LOD	<LOD	<LOD	19.56	472.8
CPPD	7	<LOD	–	<LOD	<LOD	<LOD	<LOD	15.81
77PD	26	45.91	62.61	<LOD	<LOD	37.81	165.5	295.3
∑PPDs	–	3975	4790	466.2	2538	5739	11,670	30,960
6PPD-Q	40	32.28	72.25	<LOD	<LOD	26.19	90.34	539.1
IPPD-Q	33	60.53	256.8	<LOD	<LOD	19.25	190.8	2347
DTPD-Q	1	<LOD	–	<LOD	<LOD	<LOD	<LOD	12.33
DPPD-Q	34	76.77	380.1	<LOD	<LOD	22.74	230.3	4380
CPPD-Q	7	<LOD	–	<LOD	<LOD	<LOD	20.86	49.52
77PD-Q	13	<LOD	–	<LOD	<LOD	<LOD	16.97	30.82
∑PPD-Qs	–	204.7	467.3	58.35	80.34	140.33	603.71	4451

Note: DF: detection frequency; SD: standard deviation; P25, P50, P75, and P95: 25th, 50th, 75th, and 95th percentiles, respectively; Max: maximum concentration; LOD: limit of detection; ∑PPDs: sum of the six measured PPDs; ∑PPD-Qs: sum of the six measured PPD-Qs. Data below LOD were treated as 1/2 LOD.

Overall, PPDs had significantly higher contamination levels than PPD-Qs. Among the target PPDs, 6PPD is currently the most heavily used PPD, and both 6PPD and its derivative, 6PPD-Q, have been widely reported in environmental matrices (Cao et al., 2022; Geng et al., 2025). In the present study, 6PPD was detected in 97% of the samples, with a median level of 2125 pg/g, accounting for 84% of the total median of PPDs, which is consistent with its current widespread use. In contrast, although 6PPD-Q is the oxidation product of 6PPD, it showed a lower DF of 40% and a much lower mean level of 32.28 pg/g, indicating its limited occurrence in the tested infant formula samples.

No standard analytical method has been set for the quantification of PPDs and PPD-Qs. However, analytical bias was unlikely to be the main reason why 6PPD-Q levels were much lower than 6PPD levels, because both compounds were quantified using their corresponding isotopically labelled ISs, and matrix effects and recovery losses should have been largely corrected. Nevertheless, the effectiveness of the protective agent was not independently evaluated by comparing extraction with and without antioxidant protection. Therefore, possible transformation or degradation during sample preparation cannot be completely excluded and should be further assessed in future method-optimization studies.

The lower occurrence of 6PPD-Q compared with 6PPD may be related to compound-specific stability and bioaccumulation behavior. Current evidence suggests that 6PPD-Q showed weak accumulation in biota. For example, low detection frequencies and concentrations of 6PPD-Q have been reported in mussel and fish tissues (Kuo et al., 2025). Another study on estuarine species also reported low concentrations and low bioaccumulation factors for 6PPD-Q (log*BAF* = 1.3–1.9) (Wei et al., 2024). These findings may partly explain why 6PPD-Q was much less abundant than 6PPD in infant formula.

The lower occurrence of 6PPD-Q may also be related to the sources of PPDs and PPD-Qs in infant formula, while the sources remain unclear. One possible source is environmental exposure of dairy animals followed by transfer into raw milk. Recent reviews have demonstrated the widespread occurrence of PPDs and PPD-Qs in environmental matrices (Ihenetu et al., 2024; Wang et al., 2026; Yi et al., 2025). Some studies have reported higher levels of 6PPD than 6PPD-Q in water, sediment, or soil (Cao et al., 2022; Zhu et al., 2024), which may contribute to greater environmental availability of 6PPD. However, other studies have reported different PPD/PPD-Q profiles in environmental media, suggesting that the transformation and persistence of PPDs and PPD-Qs are affected by multiple factors, such as environmental conditions, oxidation processes, and compound-specific half-lives (Geng et al., 2025; Zhang, Yan, et al., 2024).

Other sources should also be considered. Infant formula contains lipid-rich ingredients, especially vegetable oils, which are added to adjust the fatty acid composition of the product. These ingredients may themselves contain PPDs or PPD-Qs, or may favor the retention of lipophilic contaminants introduced during production. The log*K*_*ow*_ of 6PPD (4.47) is higher than that of 6PPD-Q (3.98), indicating stronger lipophilicity (Cao et al., 2022). Therefore, 6PPD may have a greater tendency to partition into lipid-rich matrices, which could contribute to its higher levels in infant formula. However, lipophilicity may affect partitioning not only during biological transfer into milk, but also during formula production (ingredient mixing, packaging contact, etc.). In addition, rubber-derived compounds may enter foods through contact with food-processing or packaging materials. Infant formula manufacturing involves many processing steps, and rubber-containing parts such as gaskets, seals, tubing, and conveyor components may provide potential contact sources and contribute to contamination for lipophilic compounds. These possibilities are consistent with the finding that no significant difference was observed between domestic and imported formulas (see Section 3.2 in detail), despite their different geographic origins and supply chains.

Overall, the occurrence of PPDs/PPD-Qs in infant formulas, as well as the higher levels of 6PPD than 6PPD-Q, cannot be attributed solely to transfer through cow milk, but may instead reflect more widespread sources of these compounds associated with raw materials and ingredients, processing equipment, packaging, or a combination of them. Further studies analyzing paired raw milk, formula ingredients, processing-contact materials, packaging materials, and final products are needed to identify the major sources of PPDs and PPD-Qs and reasons for the higher occurrence of 6PPD than 6PPD-Q.

#### 3.1.2 Other PPDs and PPD-Qs in infant formula

IPPD had the second-highest DF and contamination levels in the infant formula samples. It was detected in 74% of the 141 samples, with mean and median levels of 378.5 and 79.79 pg/g, respectively. As only 6PPD and IPPD had DFs of >50%, the correlation between them was assessed. Spearman analysis showed that levels of 6PPD and IPPD were significantly and positively correlated (*r* = 0.376, *P* < 0.01), suggesting these two compounds showed partial co-occurrence or may share some related contamination pathways. Similar to the comparison between 6PPD and 6PPD-Q, the DF (33%) and mean level (60.53 pg/g) of IPPD-Q were low. The Log*K*_*ow*_ values of IPPD and IPPD-Q are 3.28 and 2.58, respectively, and therefore the stronger lipophilicity of IPPD might be a reason for its higher DF and levels than IPPD-Q in the formula samples. Besides our study, IPPD also had a high DF in Wu's study on PPDs in human milk, in which IPPD was even the predominant PPD (Wu et al., 2024). However, in 120 human milk samples tested by (Liang et al., 2024), IPPD showed a low DF of only 15%, and IPPD-Q was not detected. In the environment, IPPD showed high DFs in various media, including water, soil, and air particles; in some studies, it was even the most abundant PPD (Geng et al., 2025; Zhang, Yan, et al., 2024; Zhu et al., 2024). Overall, the high DF of IPPD in the infant formulas, together with its high DFs and concentrations observed in various environmental media, suggested that it is another widely used PPD at present.

Other PPDs and PPD-Qs all had low DFs and concentrations. DTPD had a relatively high DF of 38%, and its mean level was 42.41 pg/g. However, DTPD-Q was barely detected. DPPD and CPPD were detected in <10% of the samples. CPPD-Q, the derivative of CPPD, also had very low DF and mean concentration. However, DPPD-Q, the derivative of DPPD, had a relatively high DF (34%) and mean concentration (76.77 pg/g). 77PD had a DF of 26% and a mean level of 45.91 pg/g, while its derivative, 77PD-Q, showed much lower DF (13%). Overall, PPDs generally showed higher DFs and concentrations than those of PPD-Qs in the infant formulas, and the higher lipophilicity of PPDs might be a reason. In current references, these four PPDs, namely DPPD, CPPD, 77PD, and DTPD, and their derivatives, all showed medium or high DFs in various environmental matrices; however, they normally account for a small portion of the total PPDs and PPD-Qs (Cao et al., 2022; Geng et al., 2025; Zhang, Yan, et al., 2024; Zhu et al., 2024). Previous studies based on food web investigation showed that PPDs and PPD-Qs tend to be distributed in sediment rather than biota, and they exhibited trophic dilution or weak trophic magnification (Zeng et al., 2025), which might explain the low DFs and concentrations of these PPDs/PPD-Qs in our study, although they had high DFs in the environment.

### 3.2 Distribution of 6PPD and IPPD in formulas with different origins and stages

**Fig. 1:**
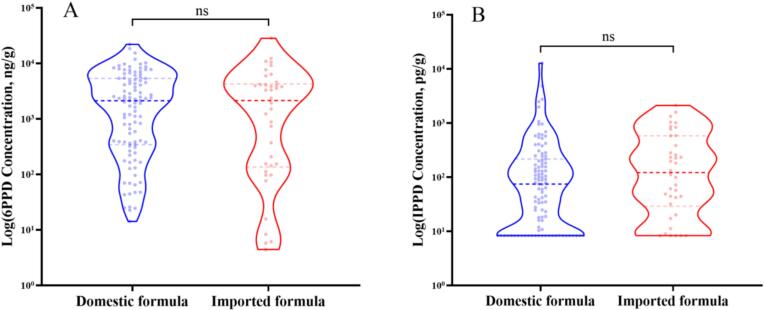
A comparison of 6PPD and IPPD concentrations between domestic and imported formulas (ns: no significant difference).

In this section, only 6PPD and IPPD were included in the analysis because they had DFs of higher than 50%. Among the tested 141 samples, 103 of them were produced in China, and the remaining 38 samples were imported from New Zealand, France, Australia, the Netherlands, Ireland, Germany, and Canada. Using the Mann-Whitney *U* test, no significant difference was observed in concentrations of 6PPD and IPPD between domestic and imported formulas (Fig. 1), indicating that the contamination of these two chemicals is ubiquitous.

**Fig. 2:**
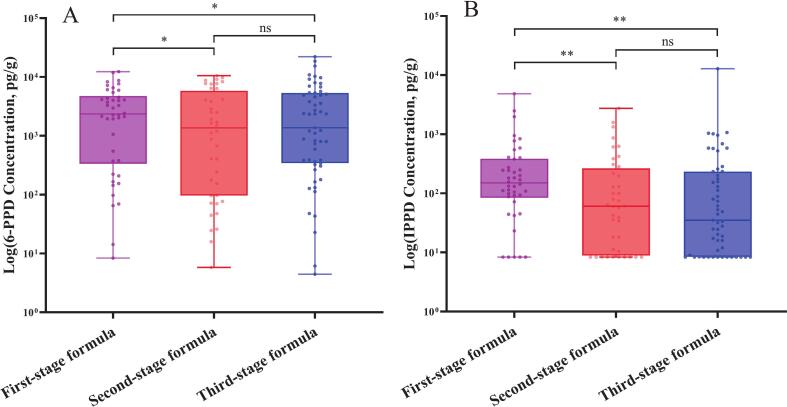
Comparison of concentrations of 6PPD (A) and IPPD (B) across formulas from the first- to third-stages. The boxes cover the 25th and 75th percentiles, the lines within the boxes indicate the median values, and the ends of the bars represent the minimum and maximum values. Ns means no significant difference, and one and two asterisks indicate significant differences at *P* < 0.05 and < 0.01, respectively.

In the tested 141 samples, there were 42 first-stage formula samples (for infants aged 0–6 months), 40 s-stage (for 7–12 months), and 55 third-stage (for 13–36 months). Since only four fourth-stage formula samples were tested, they were included in the overall occurrence analysis but were not included in stage-specific statistical comparisons and EDI calculations because of the small sample size. For 6PPD, its median levels in the first- to third-stage formula samples were 2348, 1355, and 1379 pg/g, respectively. Further analysis showed that the levels of 6PPD in the first-stage formula were significantly higher than those of both the other two groups, and no significant difference was observed between the second- and third-stage formulas (Mann-Whitney U test, *P* < 0.05) (Fig. 2A). For IPPD, its median levels in the first- to third-stage formula samples were 149.7, 60.45, and 35.06 pg/g, respectively. The median level of IPPD kept decreasing from the first-stage formula to the third-stage formula. Similar to the occurrence of 6PPD, statistical analysis showed that IPPD levels in the first-stage formulas were significantly higher than those in the other two groups (Fig. 2B).

Protein, lipid, and total carbohydrates are the main nutrients in infant formula, while their proportions vary in formulas at different stages, aiming to satisfy the nutritional needs of infants of different ages. According to the *Chinese National Food Safety Standard for Infant Formula (GB 10765–2021)*, lipid is exceptionally important for infants aged 0–6 months, and the lipid content in the first-stage formula must be higher than 1.05 g/100 kJ. For the second- and third-stage formula, the lipid content can be reduced to 0.84 g/100 kJ. That is, the first-stage formula has higher lipid content than the other two stages of formula. Since both 6PPD and IPPD are lipophilic, their higher levels in first-stage formulas may be partly related to the higher lipid content required for this formula stage. In a previous study testing 6PPD and 6PPD-Q in fish and honey, these two analytes were detected only in fish, which suggested that 6PPD and 6PPD-Q tend to accumulate in high-fat biota (Ji et al., 2022). The abundance of other lipophilic contaminants in first-stage infant formulas has been reported. In our previous study, organophosphate esters (OPEs) were tested in infant formulas, and chloride OPEs presented significantly higher levels in the first-stage formulas (Chen et al., 2022). A study concerning polychlorinated naphthalenes (PCNs) also reported a clear decreasing trend of PCN levels in infant formulas from the first- to third-stage (Qi et al., 2024). Both Cl-OPEs and PCNs have high Log*K*_*ow*_ and strong lipophilicity (Yao et al., 2025), which might contribute to their higher contents in first-stage formulas. However, actual lipid contents of individual samples were not measured in the present study. Therefore, the above explanation should be regarded as a hypothesis rather than direct evidence. Future studies should measure or compile product-specific lipid contents and evaluate their correlation with PPD and PPD-Q concentrations.

### 3.3 Comparison with other food surveys on PPDs and PPD-Qs

Although PPDs and PPD-Qs have been identified in foods, current knowledge is largely limited to 6PPD and 6PPD-Q, while information on other PPDs/PPD-Qs remains scarce. Two studies conducted in Switzerland tested 6PPD and 6PPD-Q in vegetable and fruit samples, which reported low DFs of (< 15%) and concentrations (< 10 ng/g), respectively (Breider et al., 2025; Sartori et al., 2026). Given that 6PPD-Q is well-known for its high toxicity to salmon, current studies pay more attention to the occurrence of PPDs/PPD-Qs in fish and aquatic foods. A Chinese study tested 6PPD and 6PPD-Q in ten types of fish, and they were detected only in snakehead, weever, and Spanish mackerel fish (Ji et al., 2022). Another Chinese study tested 95 aquatic foods, including shrimp, fish, eel, and octopus, while only shrimp from aquafarms tested positive for 6PPD and IPPD, with DFs of 33.3% for 6PPD and 16.7% for IPPD, respectively, and levels of 0.06–1.59 ng/g (Zhang, Wang, et al., 2024). A study conducted in the USA measured 6PPD-Q in fish fillets, whole fish homogenates, mussels, and whale blubber, and 6PPD-Q was detected in 15 of 19 samples with low concentrations (0.05–0.25 ng/g ww) (Kuo et al., 2025). Overall, available food-monitoring studies remain limited and have mainly focused on 6PPD and 6PPD-Q; moreover, these studies report low DFs of PPDs and PPD-Qs in foods, and their levels are in the range of pg/g to ng/g, indicating limited entry of PPDs and PPD-Qs to food chains, although they are widespread in environmental matrices.

### 3.4 Comparison between PPDs/PPD-Qs and other contaminants in infant formulas

**Fig. 3:**
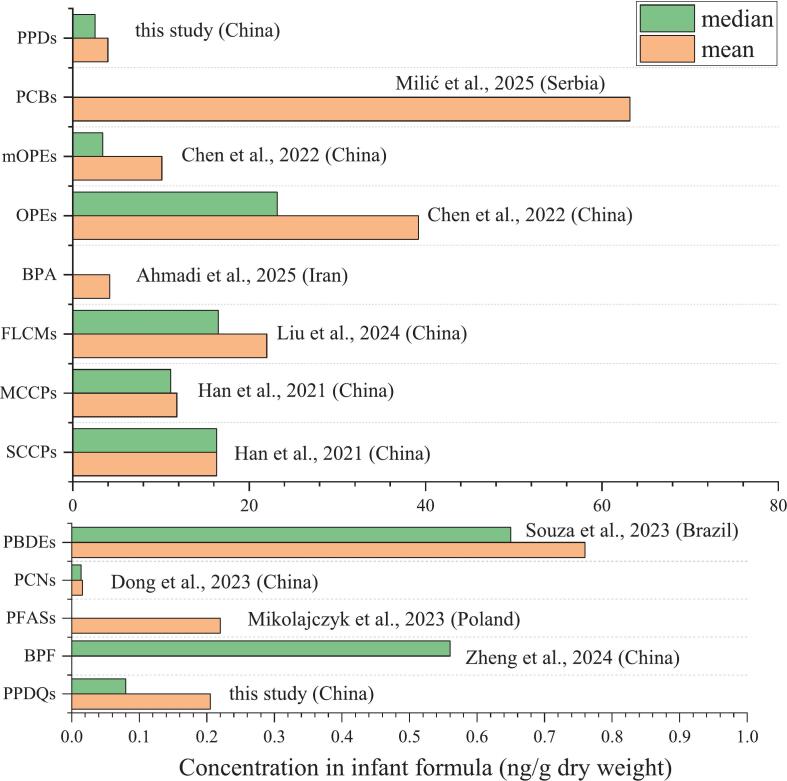
A comparison between levels of PPDs/PPD-Qs and other contaminants in infant formulas.

Environmental contaminants have been frequently detected in infant formulas in recent studies, indicating their wide presence in both the environment and biota. Using data reported in the past few years, we compared levels of PPDs/PPD-Qs with some emerging and legacy contaminants found in cow milk-based infant formulas was conducted and illustrated in Table 3 and Fig. 3. Because the included studies differed in analytical method, sample matrix, and compounds, this comparison is intended only to provide a broad contextual overview of concentration ranges rather than a direct ranking of contamination severity or health risk.

**Table 3:** Comparison on concentrations of various contaminants in infant formulas (ng/g powder).

Compound	Year	Area	Sample size	Mean	Median	Range	Note	Reference
PPDs	2024	China	141	3.975	2.538	0.081–30.96	sum of 6 PPDs	This study
PPD-Qs	2024	China	141	0.205	0.08	0.058–4.451	sum of 6 PPD-Qs	This study
PCNs	2019–2020	China	72	0.016	0.014	0.008–0.03	sum of 75 PCNs	(Dong et al., 2023)
PCBs	–	Serbia	8	63.17	–	18.08–146.14	sum of 18 PCBs	(Milić et al., 2025)
OPEs	2021	China	75	39.2	23.2	0.79–159	sum of 14 OPEs,	(Chen et al., 2022)
mOPEs	2021	China	75	10.1	3.39	0.258–74.2	sum of 5 mOPEs,	(Chen et al., 2022)
PBDEs	–	Brazil	40	0.76	0.65	0.33–1.62	sum of 7 PBDEs	(Souza et al., 2023)
FLCMs	2019	China	103	22	16.5	1.26–126	sum of 38 FLCMs	(Liu et al., 2024)
BPA	–	Iran	27	4.2	–	2.98–5.1	–	(Ahmadi et al., 2025)
BPF	2020	China	54	–	0.56	ND-4.48	–	(Zheng et al., 2024)
SCCPs	–	China	6	16.3	16.3	7.41–54.2	–	(Han et al., 2021)
MCCPs	–	China	6	11.8	11.1	3.04–20.9	–	(Han et al., 2021)
PFASs	2022	Poland	6	0.22	–		sum of 14 PFASs	(Mikolajczyk et al., 2023)

Note: PPDs: *p*-phenylenediamines; PPD-Qs: *p*-phenylenediamine quinones; PCNs: polychlorinated naphthalenes; PCBs: polychlorinated biphenyls; PBDEs: polybrominated diphenyl ethers; OPEs: organophosphate esters; mOPEs: organophosphate diester metabolites; BPA: bisphenol A; BPF: bisphenol F; FLCMs: fluorinated liquid-crystal monomers; SCCPs: short-chain chlorinated paraffins; MCCPs: medium-chain chlorinated paraffins; PFASs: *per*- and polyfluoroalkyl substances.

Levels of all the included contaminants were in the range of pg/g to ng/g, while large variations were observed. Levels of PPDs and PPD-Qs in our study, and most emerging contaminants such as OPEs and their metabolites (mOPEs), were still significantly lower than the levels of a legacy contaminant, namely polychlorinated biphenyls (PCBs), even if PCBs have been totally banned for a long time. The high historical production and application amounts of PCBs, as well as their strong environmental persistence and bioaccumulation, might be the main reasons for its abundance (Milić et al., 2025). However, levels of PPDs were higher than those of polybrominated diphenyl ethers (PBDEs) and PCNs, two groups of flame retardants or lubricants that were once widely used but have now been banned (Dong et al., 2023; Souza et al., 2023).

Compared with emerging contaminants that have recently received considerable attention, including OPEs and mOPEs, bisphenol analogues, *per*- and polyfluoroalkyl substances (PFAS), chlorinated paraffins (CPs), and fluorinated liquid-crystal monomers (FLCMs), levels of PPDs were lower than those of OPEs and mOPEs (Chen et al., 2022), CPs (Han et al., 2021), FLCMs (Liu et al., 2024), and bisphenol analogues (Ahmadi et al., 2025), whereas levels of all these chemicals were at the ng/g level. PFASs, well-known as “forever chemicals”, presented very low concentrations in infant formulas at the pg/g level, similar to those of PPD-Qs (Mikolajczyk et al., 2023). Overall, in infant formulas, the contamination levels of PPDs and PPD-Qs were lower than most well-known environmental contaminants. As we mentioned above, PPDs and PPD-Qs tend to be distributed in sediment rather than in biota, and they have been reported to exhibit trophic dilution or weak trophic magnification (Zeng et al., 2025), which may partly explain their relatively low levels in infant formulas. However, since data on PPDs and PPD-Qs in biota and foods are scarce, given that they are highly toxic to some biota, monitoring of their occurrence in foods and associated health risks should continue.

### 3.5 Daily PPD/PPD-Q intake via formula feeding for the Chinese infants

Based on concentrations of PPDs and PPD-Qs in the formula samples, the average exposure scenario (AES) and high exposure scenario (HES) were calculated according to the mean and 95th percentile concentrations, respectively. It should be noted that values for non-detected samples were replaced by 1/2LOD during the calculation, and therefore, the calculated EDIs for those with low DFs may overestimate actual intakes.

As shown in Table 4, for Chinese infants aged 0–6 months, when results of the undetected samples were replaced by 1/2LOD, the EDI_AES_ and EDI_HES_ of ∑PPDs via formula feeding were 65.59 and 192.5 ng/kg bw/day, respectively. Considering the high DFs of 6PPD and IPPD, these two compounds were the dominant contributors to total EDIs. Under the AES, EDIs of 6PPD and IPPD were 57.41 and 6.24 ng/kg bw/day, respectively; while under the HES, their EDIs increased to 174.2 and 22 ng/kg bw/day, respectively. The high EDI values under the HES indicated that newborns may be simultaneously exposed to both contaminants through formula feeding, which warrants further evaluation of potential health risks under high-exposure scenarios. EDIs of the other four PPDs and all PPD-Qs were much lower due to the low DFs and concentrations, with values <4 ng/kg bw/day even under the HES.

**Table 4:** EDIs of PPDs and PPD-Qs via formula consumption for the Chinese infants aged 0–36 months (ng/kg bw/day).

Compounds	0–6 months		7–12 months		13–36 months	
	EDI_AES_	EDI_HES_	EDI_AES_	EDI_HES_	EDI_AES_	EDI_HES_
6PPD	57.41	174.23	42.74	129.70	25.13	76.26
IPPD	6.24	22.00	4.65	16.38	2.73	9.63
DTPD	0.70	2.27	0.52	1.69	0.31	0.99
DPPD	0.33	0.32	0.25	0.24	0.15	0.14
CPPD	0.14	0.16	0.11	0.12	0.06	0.07
77PD	0.76	2.73	0.56	2.03	0.33	1.20
∑PPDs	65.59	192.53	48.82	143.32	28.71	84.27
6PPD-Q	0.53	1.49	0.40	1.11	0.23	0.65
IPPD-Q	1.00	3.15	0.74	2.34	0.44	1.38
DTPD-Q	0.14	0.14	0.10	0.10	0.06	0.06
DPPD-Q	1.27	3.80	0.94	2.83	0.55	1.66
CPPD-Q	0.29	0.34	0.21	0.26	0.12	0.15
77PD-Q	0.15	0.27	0.12	0.20	0.07	0.12
∑PPD-Qs	3.38	9.96	2.51	7.42	1.48	4.36

Note: EDI: estimated daily intake; AES: average exposure scenario; HES: high exposure scenario; PPDs: *p*-phenylenediamines; PPD-Qs: *p*-phenylenediamine quinones; ∑PPDs: sum of the six measured PPDs; ∑PPD-Qs: sum of the six measured PPD-Qs. EDI_AES_ was calculated based on the mean concentration of the analyte, and EDI_HES_ was calculated based on the 95th percentile concentration. Non-detected values were replaced with 1/2 LOD.

For infants aged 7–12 months, EDIs of ∑PPDs and ∑PPD-Qs under the AES were 48.82 and 2.51 ng/kg bw/day, respectively; while for infants aged 13–36 months, these EDIs decreased to 28.71 and 1.48 ng/kg bw/day, respectively. These results indicate that the EDIs of both PPDs and PPD-Qs decreased with increasing age, because the proportion of infant formula in infants' diet decreases with age, while body weight rises. However, intakes of PPDs and PPD-Qs via the consumption of complementary foods for infants aged >6 months should be considered in future studies.

For compounds with low DFs, the substitution of non-detected values with 1/2 LOD may introduce positive bias into EDI estimates. To evaluate this uncertainty, a sensitivity analysis was conducted using three substitution assumptions: ND = 0, ND = 1/2 LOD, and ND = LOD. Under these three scenarios, EDIs of ∑PPDs under the AES were 64.89, 65.59, and 66.28 ng/kg bw/day, respectively. Only a slight difference was observed for EDIs of ∑PPDs because 6PPD and IPPD had high DFs and dominated the total EDI. However, for the four PPDs with DFs < 40%, including DPPD, CPPD, DTPD, 77PD, EDIs of ∑_4_PPDs under these three scenarios were 1.27, 1.93, and 2.6 ng/kg bw/day, respectively. That is, EDI estimates for low-DF analytes were sensitive to the substitution method and their EDIs should be regarded as screening-level estimates rather than precise exposure values.

Exclusive breastfeeding is generally recommended for infants aged 0–6 months, while only two studies have reported PPDs/PPD-Qs in human milk to date. Based on measurements of 120 human milk samples, Liang et al. reported that the median EDIs of 6PPD via breastfeeding were 0.615, 0.574, 0.451, and 0.34 ng/kg bw/day for infants aged <1 month, 1–3 months, 3–6 months, and 6–12 months, respectively; and upper-bound EDIs calculated using 95th percentile concentrations for those infants were 7.07, 6.59, 5.18, and 3.91 ng/kg bw/day, respectively (Liang et al., 2024). By testing 15 samples, Wu et al. reported that the average EDIs of total PPDs and PPD-Qs for newborns (< 1 month) were 2300 and 1600 ng/kg bw/day, respectively, and the 95th percentile EDI of PPDs+PPD-Qs was 9400 ng/kg bw/day (Wu et al., 2024). The two available human-milk studies reported markedly different results. Such discrepancies may reflect differences in sample size, region, exposure background, and analytical sensitivity. Moreover, such discrepancies suggest that analytical comparability and reliability remain important challenges for PPD/PPD-Q determination in milk matrices and lead to substantial uncertainty in exposure estimates.

Our estimated intake was higher than that reported by (Liang et al., 2024) but much lower than that of (Wu et al., 2024), and we are unable to determine whether infants receive higher exposure through formula feeding or breastfeeding. Nevertheless, it would be inappropriate to conclude that breastfeeding or formula feeding should be restricted based solely on this comparison. Breastfeeding generally provides greater benefits for infant growth and development than formula feeding, while for mothers who cannot produce sufficient breast milk or are unable to breastfeed for any reason, infant formula is the best choice. Moreover, the comparison highlights the need to investigate the potential sources and routes of PPD/PPD-Q contamination to reduce exposure via both breast- and formula feeding.

Only one study has reported EDIs of PPDs and PPD-Qs via food consumption for adults. With the consumption of fruit, root, and leafy vegetables, dietary intakes of 6PPD and 6PPD-Q in Swiss adults were 0–42.3 and 0–18.7 ng/person/day, respectively (Breider et al., 2025). Compared to our result, the body burden of these two compounds on infants was significantly higher due to low infant body weight and relatively high levels of 6PPD in formulas, which reminds the necessity to further assess infants' health risks caused by PPD/PPD-Q exposure.

### 3.6 Comparison with other exposure pathways

In addition to food consumption, water, dust, and air are also important exposure pathways for environmental contaminants. It has been reported that water exposure accounted for 82.5% of human exposure to PPDs and PPD-Qs (Geng et al., 2025). In a nationwide survey of PPDs and PPD-Qs in the riverine waters of China, the median EDIs of ∑PPDs and ∑PPD-Qs via water exposure for children (aged 5–6 years) were 1.07 and 3.39 ng/kg bw/day, respectively (Geng et al., 2025). However, in Geng's study, IPPD and IPPD-Q were predominant, while 6PPD accounted for only a small portion of the total PPDs. Another Chinese national survey on road dust reported that the EDIs of ΣPPDs (mainly 6PPD) and 6PPD-Q via dust ingestion were 0.143 and 0.076 ng/kg bw/day, respectively; while via dermal contact with dust were 0.021 and 0.011 ng/kg bw/day, respectively (Zhang, Yan, et al., 2024). By measuring PPDs and PPD-Qs in water, air, and soil, a study conducted in Hong Kong (China) reported that the total daily intakes of PPDs and PPD-Qs for children were 4.85 and 7.3 ng/kg bw/day, respectively, and oral ingestion of roadside soil dust contributed the most to the total EDI. Moreover, in the Hong Kong study, levels of 6PPD and 6PPD-Q in soil were at least one order of magnitude higher than other PPDs and PPD-Qs (Cao et al., 2022). Based on the limited available data, formula feeding may be an important dietary exposure pathway for infants. Moreover, although profiles of PPDs and PPD-Qs vary among studies, 6PPD and IPPD are often major contributors.

### 3.7 Health risk assessment and further research suggestions

Reference doses (RfDs) for PPDs and PPD-Qs have not yet been established for humans. Therefore, only a preliminary screening-level risk assessment was performed in this study. According to the harmonised classification and labeling (CLH) report for 6PPD released by the European chemicals agency (ECHA) in 2023 (https://echa.europa.eu), a clear target organ of 6PPD after repeated oral exposure is the liver, and a conservative NOAEL of 4 mg/kg bw/day was derived from a 28-day oral rat study based on changes in liver weight. In the absence of an official RfD, a provisional oral RfD was calculated using a general reference-dose approach, in which the point of departure (the NOAEL) is divided by uncertainty factors. A total uncertainty factor of 1000 was applied, including 10 for interspecies extrapolation, 10 for intraspecies variability, and an additional 10 to account for the incomplete toxicological database, especially the lack of infant-specific toxicokinetic and toxicodynamic data. Thus, the provisional oral RfD for 6PPD was calculated as 4 μg/kg bw/day

The estimated EDIs of 6PPD via formula feeding were lower than this provisional oral RfD, suggesting that 6PPD exposure from infant formula alone may not pose an obvious health concern under the exposure scenarios considered. However, this conclusion should be interpreted cautiously because the reference value is provisional and conservative. In addition, no NOAELs or RfDs are available for most other PPDs and PPD-Qs, and relative potency factors or toxic equivalency factors haven't been established. Therefore, cumulative risk from co-exposure to multiple PPDs and PPD-Qs could not be quantitatively evaluated in the present study. Given their co-occurrence and partially overlapping toxicological endpoints (Song et al., 2024), mixture-risk assessment should be considered a priority in future studies.

Additionally, toxicokinetic information on PPDs and PPD-Qs remains limited, particularly for infants. However, available mammalian evidence indicates that these compounds can be absorbed after oral exposure and distributed to internal tissues. In a mouse model, 6PPD-Q was rapidly assimilated and reached peak concentrations in blood and major organs within 1 h. It was mainly distributed in adipose tissue, followed by kidney, lung, testis, liver, spleen, heart, and muscle, and it penetrated the blood-brain barrier. The reported half-lives of 6PPD-Q in various organs were less than 24 h (Zhang, Cao, et al., 2024). In addition, repeated oral exposure to 6PPD and 6PPD-Q resulted in dose-dependent accumulation in mouse liver (Fang et al., 2023). These findings suggest that orally ingested PPDs/PPD-Qs may undergo gastrointestinal absorption and tissue distribution, especially to lipid-rich tissues. This is consistent with their lipophilic properties and with our observation that first-stage formula, which has higher lipid content, contained higher levels of 6PPD and IPPD. Nevertheless, because toxicokinetic data for human infants are unavailable, the present risk assessment remains preliminary and is subject to uncertainty.

## 4 Conclusion

In this study, twelve PPDs and PPD-Qs were measured in infant formulas collected in China. 6PPD and IPPD presented high DFs, while the other analytes had much lower DFs and contamination levels. Similar occurrence patterns of PPDs and PPD-Qs in foods have been observed in other surveys, suggesting limited transfer of PPDs and PPD-Qs into food chains. However, 6PPD was detected in most samples and showed relatively high concentrations, suggesting an urgent need to evaluate its health risks. First-stage formulas for infants aged 0–6 months had significantly higher concentrations of both 6PPD and IPPD than formulas for older infants, and the high lipid content of the first-stage formulas and the strong lipophilicity of these two chemicals may partly explain their abundance, which may indicate a higher exposure burden in newborns. Based on comparison with the limited available exposure studies in adults and older children, formula feeding may represent an important dietary exposure pathway for infants. Moreover, the daily intakes of PPDs and PPD-Qs via formula feeding for infants may be higher than those for adults via food consumption, which highlights the need for further assessment of infants' health risks caused by PPD/PPD-Q exposure. The estimated intake of 6PPD through formula feeding was lower than the provisional oral RfD used in this study, although this result should not be regarded as definitive evidence of safety. No RfDs or NOAELs are available for other PPDs and PPD-Qs; mixture effects were not quantitatively evaluated; and infant-specific toxicokinetic and toxicodynamic data remain unavailable, which suggests the need for further toxicological research and continued exposure monitoring. Our findings provide critical insights into the safety of infant feeding practices, highlighting the need for regulatory measures and monitoring strategies to protect vulnerable populations. By contributing data on food safety and chemical exposure, our research offers essential recommendations for parents, healthcare professionals, and policymakers to ensure the health and well-being of infants.

## CRediT authorship contribution statement

**Zifu Shao:** Writing – original draft, Methodology, Investigation. **Chunyan Gao:** Writing – review & editing, Supervision, Conceptualization. **Zhixiong Shi:** Writing – review & editing, Supervision, Funding acquisition, Conceptualization.

## Declaration of competing interest

The authors declare that they have no known competing financial interests or personal relationships that could have appeared to influence the work reported in this paper.

## Data Availability

Data will be made available on request.
